# Enzyme Kinetics and Molecular Docking Studies on Cytochrome 2B6, 2C19, 2E1, and 3A4 Activities by Sauchinone

**DOI:** 10.3390/molecules23030555

**Published:** 2018-03-02

**Authors:** Eun Chae Gong, Satya Chea, Anand Balupuri, Nam Sook Kang, Young-Won Chin, Young Hee Choi

**Affiliations:** 1 College of Pharmacy and Intergrated Research Institute for Drug Development, Dongguk University-Seoul, 32 Dongguk-lo, Ilsandong-gu, Goyang, Gyeonggi-do 10326, Korea; dmsco3901@hanmail.net (E.C.G.); satyachea168@gmail.com (S.C.); f2744@dongguk.edu (Y.-W.C.); 2 Graduate School of New Drug Discovery and Development, Chungnam National University, Daejeon 305-764, Korea; balupuri@cnu.ac.kr (A.B.); nskang@cnu.ac.kr (N.S.K)

**Keywords:** sauchinone, *Saururus chinensis*, CYP450, metabolic inhibition, herb-drug interaction, human liver microsome, docking

## Abstract

Sauchinone, an active lignan isolated from the aerial parts of *Saururus chinensis* (Saururaceae), exhibits anti-inflammatory, anti-obesity, anti-hyperglycemic, and anti-hepatic steatosis effects. As herb–drug interaction (HDI) through cytochrome P450s (CYPs)-mediated metabolism limits clinical application of herbs and drugs in combination, this study sought to explore the enzyme kinetics of sauchinone towards CYP inhibition in in vitro human liver microsomes (HLMs) and in vivo mice studies and computational molecular docking analysis. In in vitro HLMs, sauchinone reversibly inhibited CYP2B6, 2C19, 2E1, and 3A4 activities in non-competitive modes, showing inhibition constant *(K*_i_) values of 14.3, 16.8, 41.7, and 6.84 μM, respectively. Also, sauchinone time-dependently inhibited CYP2B6, 2E1 and 3A4 activities in vitro HLMs. Molecular docking study showed that sauchinone could be bound to a few key amino acid residues in the active site of CYP2B6, 2C19, 2E1, and 3A4. When sibutramine, clopidogrel, or chlorzoxazone was co-administered with sauchinone to mice, the systemic exposure of each drug was increased compared to that without sauchinone, because sauchinone reduced the metabolic clearance of each drug. In conclusion, when sauchinone was co-treated with drugs metabolized via CYP2B6, 2C19, 2E1, or 3A4, sauchinone–drug interactions occurred because sauchinone inhibited the CYP-mediated metabolic activities.

## 1. Introduction

Herb–drug combination therapies are widely used due to their therapeutic benefit, but risks of herb–drug interactions (HDIs) have also been reported [1,2,3]. Among various HDIs, attention should be paid to pharmacokinetic interactions between co-administered herbs and drugs, because such interactions could lead to altered therapeutic efficacies and/or toxicities [4,5]. Cytochrome P450 (CYP) enzymes possess broad substrate specificities to metabolize xenobiotics (almost 90% of drugs and herbs) and endogenous compounds [6,7,8]. Many clinical cases associated with CYP inhibition by herbs have been reported, including St. John’s wort, ginko, milk thistle, cranberry, and garlic [7,8,9,10,11,12]. For example, concomitant administration of St. John’s wort with protease inhibitors in antiretroviral therapies or warfarin has increased concentrations of protease inhibitors and warfarin via inhibiting CYP3A4 and 2C9, respectively, thus changing their efficacy and safety [13]. In these regards, evaluating CYP inhibition-mediated HDIs has been strongly recommended by the United States of America Food and Drug Administration (FDA) [14,15].

*Saururus chinensis* Hort. ex Loudon (Saururaceae), Sam-baek-cho in Korean, is a plant with a long history of medical use for treating beriberi, hyperlipidemia, fever, jaundice, edema, and inflammatory disease [16]. Sauchinone, a biologically active lignan isolated from the aerial parts of *S. chinensis*, also shows anti-oxidative, anti-inflammatory, hepatoprotective, anti-cardiovascular, anti-obesity, and anti-hepatic steatosis activities [17,18,19,20,21,22]. Recently, the approval of ESP-102 as a functional food containing sauchinone from *S. chinensis* (Daewoong Bio, South Korea; [17]) shows that *S. chinensis* can be developed as a source of functional food or herbal medicine in combination therapies [18,19,20,21,22]. As a major component of *S. chinensis*, sauchinone is extensively metabolized via CYPs and is highly distributed in the liver [23], suggesting that sauchinone–drug interaction may occur via CYP inhibition. However, up to date, there is no information about sauchinone–drug interaction through CYP inhibition. Therefore, the objective of this study was to explore the inhibitory effects of sauchinone on CYPs (CYP1A2, 2A6, 2B6, 2C9, 2C19, 2D6, 2E1, and 3A4) in in vitro human liver microsomes (HLMs) and determine pharmacokinetic interactions of sibutramine, clopidogrel, or chlorzoxazone (as representative drugs metabolized by CYP2B6, 2C19/3A4, and 2E1, respectively) with sauchinone in in vivo mice. In addition, computational molecular docking analysis was performed to understand how sauchinone interacted with particular CYPs (CYP2B6, 2C19, 2E1, and 3A4).

## 2. Results

### 2.1. Reversible Inhibition (RI) of Sauchinone on CYP Activities in HLMs

**Figure 1:**
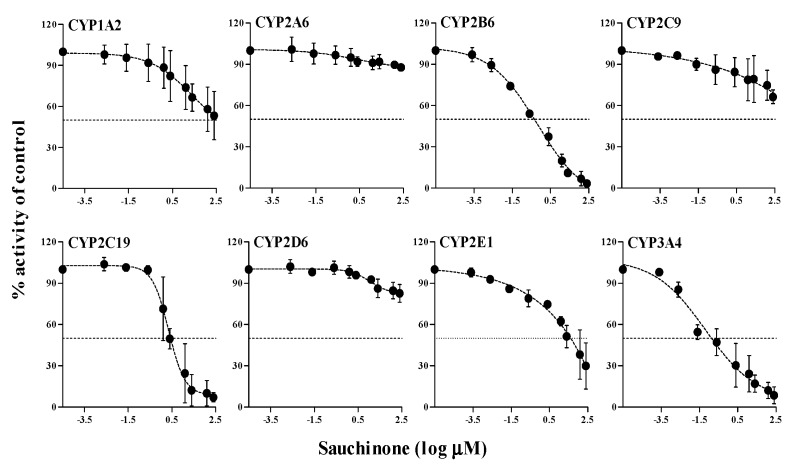
IC_50_ curves of sauchinone for RI on CYP1A2, CYP2A6, CYP2B6, CYP2C9, CYP2C19, CYP2D6, CYP2E1, and CYP3A4 in HLMs. Y-axis is expressed as the remaining percentage of activity with sauchinone compared with the control (without sauchinone).

Results for RI of sauchinone and well-known reversible inhibitors of each CYP isoform (fluvoxamine, tranylcypromine, paroxetine, sulfaphenazole, omeprazole, quinidine, 4-methylpyrazole, and ketoconazole for CYP1A2, 2A6, 2B6, 2C9, 2C19, 2D6, 2E1, and 3A4, respectively) on CYP1A2, 2A6, 2B6, 2C9, 2C19, 2D6, 2E1, and 3A4 are shown in Figure 1. Their half maximal inhibitory concentration (IC_50_) values were listed in Table 1. When sauchinone concentrations were adjusted up to 300 μM, IC_50_ values of sauchinone on CYP2B6-catalyzed bupropion hydroxylation, 2C19-catalyzed *S*-mephenytoin hydroxylation, and 3A4-catalyzed testosterone hydroxylation were 0.458, 3.60, and 0.207 μM, respectively, which were lower than 5 μM. These IC_50_ values were close to those of their well-known direct inhibitors (0.154, 6.59, 0.0845 μM of paroxetine, omeprazole, and ketoconazole, respectively). Sauchinone also inhibited 2E1-catalyzed chlorzoxazone hydroxylation with IC_50_ of 35.4 μM, similar to that of 4-methylpyrazole (35.8 μM), a well-known CYP2E1 inhibitor. The RI of sauchinone on other CYPs such as CYP1A2, 2A6, and 2D6 appeared to be negligible because IC_50_ values were over 300 μM.

### 2.2. Apparent Inhibition Constant (K_i_) Values of Sauchinone for CYP2B6, 2C19, 2E1, and 3A4 in HLMs

**Figure 2:**
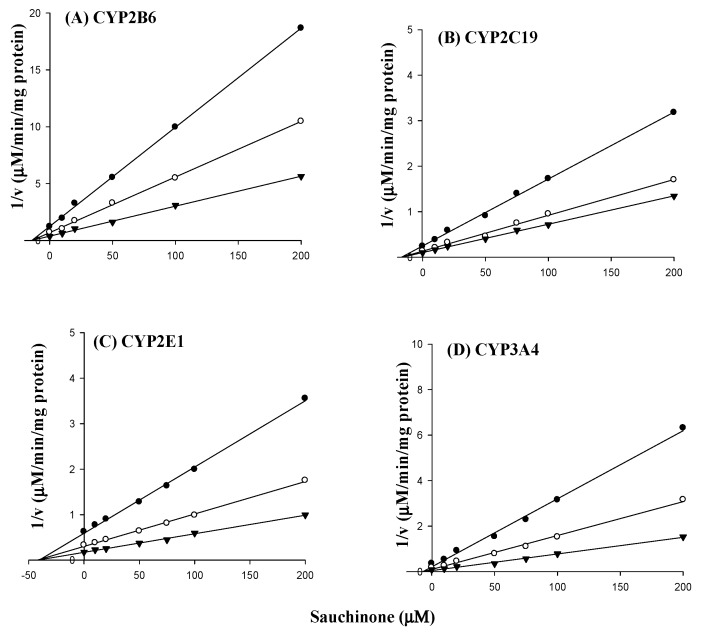
Dixon plots for inhibitory effects of sauchinone at various concentrations (●, 20 μM; ○, 50 μM; ▼, 200 μM) on CYP2B6 (**A**); 2C19 (**B**); 2E1 (**C**); and 3A4 (**D**) activities.

The enzyme kinetic assays on inhibition of CYP2B6, 2C19, 2E1, and 3A4 activities were conducted with various concentrations of sauchinone to characterize the type of RI based on the estimated IC_50_ values in RI (Table 1). As shown in Figure 2, all Dixon plots for the inhibition of CYP2B6, 2C19, 2E1, and 3A4 by sauchinone were fitted well to the noncompetitive inhibition model in visual inspection and the *K_i_* values of sauchinone for CYP2B6, 2C19, 2E1, and 3A4 are 14.3, 16.8, 41.7, and 6.84 μM, respectively.

### 2.3. Time-Dependent Inhibition (TDI) of Sauchinone on CYP Activities in HLMs

**Figure 3:**
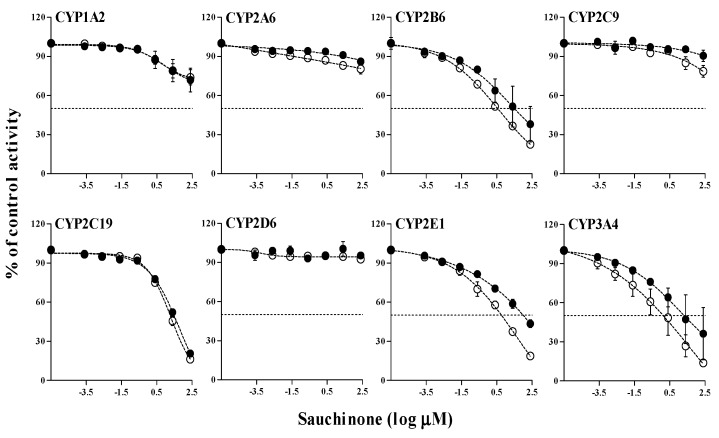
IC_50_ curves of sauchinone for TDI on CYP1A2, CYP2A6, CYP2B6, CYP2C9, CYP2C19, CYP2D6, CYP2E1, and CYP3A4 in HLMs. Y-axis is expressed as the remaining percentage of activity with sauchinone compared with the control (without sauchinone). Symbols of ‘●’ and ‘○’ represent 0 and 30-min inactivation incubation, respectively.

After pre-incubating HLMs with sauchinone and β-nicotinamide adenine dinucleotide phosphate (NADPH) for 30 min as an inactivation step, inhibitory effects of sauchinone and well-known time-dependent inhibitors of each CYP isoform (furafylline, 8-methoxypsoralen, ticlopidine, tienilic acid, fluoxetine, paroxetine, disulfiram, and verapamil for CYP1A2, 2A6, 2B6, 2C9, 2C19, 2D6, 2E1, and 3A4, respectively) were examined. Results are shown in Figure 3. Their IC_50_ values and IC_50_ shift were also listed in Table 2. In our preliminary study to determine the incubation time of TDI, 30 min of inactivation incubation for HLM with an inhibitor reduced the activity of HLM. Therefore, a shorter incubation time (10 min) was selected for incubation after pre-incubation. By calculating IC_50_ shift values of well-known time-dependent inhibitors, properties of inactivation incubation time of 30-min and incubation time for 10-min were verified. Sauchinone time-dependently inhibited CYP2B6, 2E1, and 3A4 with IC_50_ shifts in 30-min inactivation incubation of 9.28, 20.9, and 21.4-fold, respectively, compared to those without inactivation incubation. This indicated that sauchinone’s metabolites formed in 30 min of pre-incubation time might have inhibitory effects on CYP2B6, 2E1, and 3A4. It has been reported that tentative metabolites of sauchinone are formed through CYPs in the liver [23]. However, sauchinone showed no apparent inhibition on other CYPs, with IC_50_ shift less than 2-fold.

### 2.4. Molecular Docking Study

Computational modeling of CYP and ligand interactions has gained a considerable amount of attention in recent years. Sauchinone was docked inside the active sites of human CYP3A4, CYP2B6, 2C19, and CYP2E1 to explore its binding mode and interactions.

**Figure 4:**
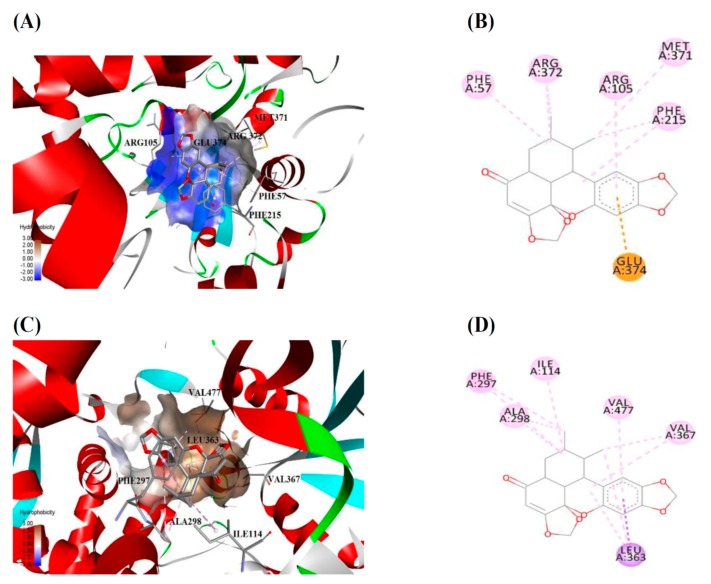

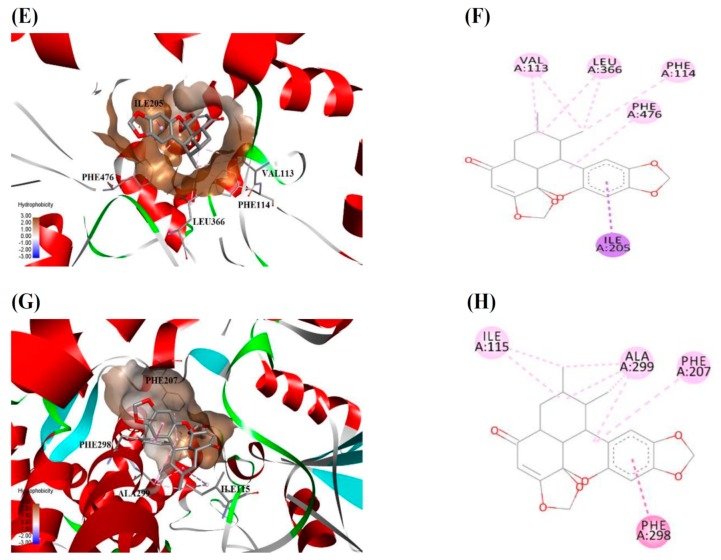
Molecular docking analysis demonstrating binding positions of sauchinone in human CYP3A4 (PDB code: 3UA1), CYP2B6 (PDB code: 3IBD), CYP2C19 (PDB code: 4GQS), and CYP2E1 (PDB code: 3GPH). Three-dimensional illustrations show interactions of sauchinone with human CYP3A4 (**A**); 2B6 (**C**); 2C19 (**E**); and 2E1 (**G**) at labelled amino acid residues. Two-dimensional diagrams display interactions of sauchinone in the active sites of CYP3A4 (**B**); 2B6 (**D**); CYP2C19 (**F**); and 2E1 (**H**). Colors of dotted lines explain types of various interactions: hydrophobic (pink), π-anion (orange), and π-sigma (purple). For color in this figure, please see web version of this article.

For human CYP3A4, sauchinone showed a high binding energy of −8.64 kcal/mol at the active site of CYP3A4 (PDB code: 3UA1). Docking results demonstrated that sauchinone was placed in the cavity enclosed by Phe57, Arg105, Arg106, Phe108, Phe215, Met371, Arg372, Leu373, Glu374, and Arg375. As shown in Figure 4A,B; Phe57, Arg105, Phe215, Met371, and Arg372 were involved in hydrophobic interactions with sauchinone, while Glu374 showed π-anion interaction with sauchinone. For human CYP2B6, sauchinone displayed a relatively low binding energy of −8.47 kcal/mol at the active site of CYP2B6 (PDB code: 3IBD). Sauchinone was positioned in the binding pocket formed by Ile101, Ile114, Phe115, Phe297, Ala298, Glu301, Thr302, Leu363, Gly366, Val367, Pro368, and Val477. As shown in Figure 4C,D, Ile114, Phe297, Ala298, Val367, and Val477 demonstrated hydrophobic interactions with sauchinone, whereas Leu363 exhibited π-sigma interaction with sauchinone. In the case of human CYP2C19, sauchinone exhibited a much lower binding energy of −7.76 kcal/mol at the active site of CYP2C19 (PDB code: 4GQS) than that for CYP2B6. Sauchinone was accommodated in the active site, surrounded by Val113, Phe114, Ile205, Asp293, Gly296, Glu300, Ile362, Ile366, and Phe476. As shown in Figure 4E,F, Val113, Phe114, Ile366, and Phe476 contributed to hydrophobic interactions with sauchinone, whereas Ile205 demonstrated π-sigma interaction with sauchinone. Sauchinone showed a very low binding energy of −1.81 kcal/mol at the active site of CYP2E1 (PDB code: 3GPH). The binding pocket of sauchinone in CYP2E1 was composed of Ile115, Phe203, Phe207, Phe298, Ala299, Glu302, Thr303, Leu363, Val364, Leu368, Phe478, and Gly479. Hydrophobic interactions were merely responsible for the binding of sauchinone to CYP2E1. Active site residues such as Ile115, Phe207, Phe298, and Ala299 participated in hydrophobic interactions between CYP2E1 and sauchinone (Figure 4G,H).

### 2.5. Drug Interactions between Sibutramine, Clopidogrel, or Chlorzoxazone and Sauchinone in Mice

**Figure 5:**
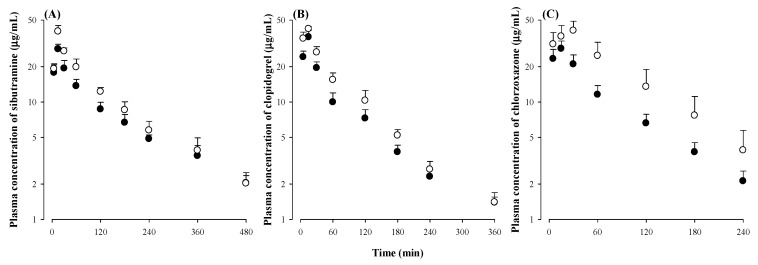
Plasma concentrations of sibutramine (**A**); clopidogrel (**B**); or chlorzoxazone (**C**) after its oral administration with vehicle (●) and sauchinone (○). The number of mice were five for each group.

Pharmacokinetic interactions between sauchinone and drugs mainly metabolized by CYP2B6, 2C19, 2E1, and/or 3A4 were evaluated in vivo using mice. Sibutramine, clopidogrel, and chlorzoxazone were chosen as representative drugs mainly metabolized by CYP2B6, CYP2C19/3A4, and CYP2E1 [24,25,26] to provide examples of sauchinone and drug interactions through CYP2B6, CYP2C19/3A4, and CYP2E1 inhibitions to be consistent with in vitro results. Moreover, a combination of sibutramine or clopidogrel with herbal supplements or foods having activities against metabolic disorders have been frequently reported [25,26]. After oral administration of sibutramine, clopidogrel, or chlorzoxazone with sauchinone to mice, the mean arterial plasma concentration–time profiles of sibutramine, clopidogrel, or chlorzoxazone were determined. Results are shown in Figure 5 and their relevant pharmacokinetic parameters are listed in Table 3. In mice receiving oral administration of sibutramine and sauchinone together, the area under the plasma concentration–time curve from time zero to the last measured time to infinity (AUC), peak plasma concentration (*C*_max_), terminal half-life (t_1/2_), and clearance/absolute bioavailability (CL/*F*) of sibutramine were significantly greater (by 23.6%), higher (by 41.5%), smaller (by 16.2%), and slower (by 19.0%), respectively, than those in mice receiving sibutramine alone. Similarly, AUC, *C*_max_, t_1/2_, and CL/*F* of clopidogrel in mice receiving clopidogrel and sauchinone together were significantly greater (by 31.0%), higher (by 17.3%), smaller (by 19.1%), and slower (by 24.7%), respectively, than those in mice receiving clopidogrel alone. In mice co-administered with chlorzoxazone and sauchinone together, AUC, *C*_max_, t_1/2_, CL/*F*, and time to reach *C*_max_ (*T*_max_) of chlorzoxazone were significantly greater (by 61.1%), higher (by 40.6%), smaller (by 9.36%), slower (by 38.0%), and greater (by 50.0%), respectively, than those in mice receiving chlorzoxazone alone.

## 3. Discussion

CYP3A4 followed by CYP2B6, 2C9, 2D6, 2C19, 2E1, 1A2, and 2A6 in sequence are still considered as the main metabolic pathways in CYP-mediated metabolic interactions, although new allelic forms of CYPs have been identified [15,27,28]. Therefore, we investigated the inhibitory effects of sauchinone on eight CYP isoforms using a cocktail probe assay in HLMs to understand potential sauchinone–drug interactions.

CYP inhibitions can be classified into RI and TDI of CYP activities [6,14,29]. RI occurs when an enzyme inhibits itself rapidly and reversibly inhibits CYP activities via rapid association and dissociation between inhibitors and CYPs. On the other hand, TDI occurs in a relatively delayed and irreversible mode through irreversible covalent binding or quasi-irreversible non-covalent tight binding of an inhibitor itself and/or its metabolites with CYPs [14,30]. TDI displays a prolonged onset of inhibition and persists even after an inhibitor is eliminated. Therefore, there is a time gap between the occurrence or disappearance of inhibition and inhibitor exposure in TDI [14,28,30]. One reason might be auto-inhibition of an inhibitor. Auto-inhibition of an inhibitor means that the metabolism of an inhibitor itself becomes slow and the inhibitor itself is present in the body longer [30]. Another reason is that TDI happens after the metabolites of an inhibitor are formed, not when an inhibitor itself is exposed [6] because an inhibitor is metabolized to a reactive intermediate which can inactivate CYPs by covalent binding [30]. TDI can bring slow-onset, cumulative, and long-lasting interaction. Therefore, evaluation of TDI is necessary to estimate the risk of adverse effects associated with inhibition of CYPs. Actually, long-term intakes of herbs tend to cause TDI which can display late inhibition of metabolic enzymes. Cases of TDI such as plumbagin, silybin, and extract of *Cassia abbreviata* have been reported in HDIs [27,31,32]. Particularly high dose administration of herbs (e.g., g unit of administered herbs) can easily inhibit CYPs. Repeated long-term use of herbs may cause irreversible or quasi-irreversible binding to CYPs, leading to TDI-mediated HDIs [33]. Thus, the inhibitory effects of sauchinone on CYPs are explored in RI and TDI together using in vitro assays.

As shown in Table 1, sauchinone strongly inhibited CYP3A4 > 2B6 > 2C19 in sequence and moderately inhibited CYP2E1. There was negligible change in metabolic rate with increasing sauchinone concentrations up to 300 μM (our unpublished data). This could be due to saturation of the enzyme. To predict IC_50_ values of sauchinone for the inhibition of CYP activities via graphing, up to 300 μM of sauchinone was used because sauchinone concentrations used in in vitro can be different from in vivo physiological levels. Hence, the pharmacokinetic interaction of sauchinone with drugs in vivo were necessary to conduct considering physiological levels of sauchinone.

To explore how CYP enzymes interacted with their substrates and inhibitors, inhibition kinetic studies were performed. As shown in Figure 2, sauchinone inhibited CYP2B6, 2C19, 2E1, and 3A4 in non-competitive manners, similar to results obtained from RI. The difference between IC_50_ and *K_i_* values could be due to different experimental conditions such as incubation time, concentrations of substrate and sauchinone, and protein amounts. However, the order of inhibitory effect of sauchinone on CYP isoforms was the same: sauchinone strongly inhibited CYP3A4 > 2B6 ≒ 2C19 and moderately inhibited CYP2E1. Non-competitive inhibition by sauchinone of CYP2B6, 2C19, 2E1, and 3A4 indicated that sauchinone might be able to reduce the activities of CYP2B6, 2C19, 2E1, and 3A4 by binding to an allosteric site of the CYPs without interfering with binding of a substrate to the active site of the CYP. As a result, metabolic clearance of a substrate was decelerated due to the inhibiting effect of sauchinone, similar to results of a previous report [3].

Interestingly, metabolites of sauchinone via oxidation, di-oxidation, methylation, demethylation, dehydrogeneation, or bis-glucuronidation in S9 fractions of liver and intestine have already been reported [23]. These results suggest that the CYP-mediated metabolic pathway of sauchinone might intervene in CYP activities, leading to auto-inhibition of sauchinone and/or reduction of metabolism of co-administered drugs through CYPs. Metabolites of sauchinone formed through CYPs can also interact with CYPs, causing sauchinone–drug interaction in a TDI manner. In this study, sauchinone time-dependently inhibited CYP2B6, 2E1, and 3A4 based on the shift of IC_50_ values, as shown in Table 2.

To further understand interactions between sauchinone and CYP2B6, 2C19, 2E1, or 3A4, molecular docking analysis was performed. Sauchinone showed the highest binding energy (−8.64 kcal/mol) at the active site of CYP3A4 with the lowest *K*_i_ (6.84 µM) against CYP3A4 among four CYPs (CYP2B6, 2C19, 2E1, and 3A4). Simulation results of the present study predicted that Phe57, Arg105, Phe215, Met371, Arg372, and Glu374 were critical residues for the binding of sauchinone to CYP3A4 (Figure 4A,B). These interactions might be accountable for the high and better inhibitory activity of sauchinone against human CYP3A4 as compared to that against human CYP2B6, CYP2C19, or CYP2E1. Previous studies have reported that Phe57, Arg105, Phe215, Arg372, and Glu374 are responsible for ligand binding in CYP3A4 [4,34,35,36,37]. Against human CYP2B6, relatively lower binding energy of −8.47 kcal/mol at the active site of CYP2B6 matched with the slightly lower inhibition (*K_i_* = 14.30 µM) compared to CYP3A4. Residues Ile114, Leu363, Phe297, Ala298, Val367, and Val477 were found to be important residues for the binding of sauchinone to human CYP2B6 (Figure 4C,D), consistent with prior research studies suggesting that Ile114, Phe297, Ala298, Leu363, Val367, and Val477 might play important roles in ligand recognition by CYP2B6 [38,39,40]. In the case of human CYP2C19, sauchinone showed a lower binding energy of −7.76 kcal/mol at the active site, consistent with low inhibition for CYP2C19 (*K_i_* = 16.80 µM) compared to that for CYP2B6 or 3A4. As shown in Figure 4E,F, Val113, Phe114, Ile205, Ile366, and Phe476 were important for the interactions between sauchinone and CYP2C19. It has been reported that Val113, Phe114, Ile205, Ile366, and Phe476 are crucial residues for ligand binding in CYP2C19 [41,42]. Sauchinone exhibited the lowest binding energy of −1.81 kcal/mol at the active site of CYP2E1, consistent with its least inhibition against human CYP2E1 (*K_i_* = 41.70 µM). Active site residues Ile115, Phe207, Phe298, and Ala299 participated in hydrophobic interactions with sauchinone (Figure 4G,H) in agreement with previous studies reporting that Ile115, Phe207, Phe298, and Ala299 are responsible for ligand binding to CYP2E1 [43,44,45]. These results provided valuable information on structure–activity relationships between sauchinone and CYP2B6, 2C19, 2E1, and 3A4.

RI and TDI-mediated metabolic inhibition by herbs in vitro system has been accepted as a useful approach to predict changes in efficacy and toxicity of co-administered drug(s) [46]. However, inhibitory effects of herbs predicted from IC_50_ and *K_i_* values in in vitro experiments are insufficient to support in vivo results (especially at clinical levels) because various metabolic pathways and other elements besides metabolic inhibition can manage HDIs in in vivo. For example, discrepancies in HDI between in vitro and clinical results have been reported in cases of milk thistle or *Panax ginseng* with drugs [9,46]. Investigation on HDIs in humans can draw the most accurate conclusion to explain changes in efficacy or adverse reactions to co-administered drugs. However, evaluating HDI at the clinical level has risk of serious HDIs. Hence, prediction of HDIs at preclinical levels has been adopted to identify potential HDIs and underlying mechanisms along with in vitro results [9,47].

In in vivo systems, pharmacokinetic parameters such as AUC and *C*_max_ are used as the main endpoints to explain systemic exposure [9]. Based on these parameters, the ratio of AUC_i_ (AUC of a substrate with an inhibitor) and AUC_0_ (AUC of a substrate without an inhibitor) over 2 has been used to consider the occurrence of pharmacokinetic drug interaction [9,30]. The ratio of an inhibitor concentration in the liver, [I], and *K_i_* can determine whether metabolic inhibition in the liver might occur, because sufficient concentration of the inhibitor in the liver, a site containing the majority of CYPs, is a critical factor that causes metabolic inhibition. [I] can be measured in tissue distribution studies at preclinical levels. However, it is impossible to measure [I] in humans. Assuming that systemic exposure is similar to tissue exposure, the *C*_max_ of an inhibitor has been used instead of [I] [30]. Unfortunately, the hepatic concentrations or *C*_max_ of sauchinone in humans have not been reported yet. Therefore, sauchinone and drug interactions cannot be estimated in humans currently.

Drugs selected in this study have been used to treat metabolic diseases or inflammation. Co-administration of these drugs with *S. chinensis* containing sauchinone is also clinically probable due to ethnopharmacological use of *S. chinensis* (e.g., utilization for treatment of beriberi, hyperlipidemia, fever, jaundice, edema, and inflammatory disease; [16]). In rats, sauchinone increased systemic exposure of sibutramine, clopidogrel, or chlorzoxazone, representing the enhanced AUC and *C*_max_ values (Table 3). This might be due to inhibition of CYP2B6, CYP2C19/3A, or CYP2E1-mediated clearance of sibutramine, clopidogrel, or chlorzoxazone by sauchinone. Comparable GI_24h_ values of these drugs between treatments with and without sauchinone indicated that sauchinone did not affect absorption of sibutramine, clopidogrel, or chlorzoxazone (our unpublished data).

## 4. Materials and Methods 

### 4.1. Chemicals and Reagents

Sauchinone (over 98% purity) was provided by Professor Chin Y.-W. (Pharmacognosy Laboratory of Dongguk University, Seoul, Korea) according to the modification of previously reported method [48]. Hydroxybupropion, 4-hydroxymephenytoin, 6-hydroxychlorzoxazone, *S*-mephenytoin, and pooled human liver microsomes from a mixed pool of 50 donors (HLMs; BD Ultra Pool HLM 50, cat. 452156) were purchased from BD Gentest Co. (Woburn, MA, USA). Acetaminophen, chlorzoxazone, clopidogrel, coumarin, furafylline, 6α-hydroxytestosterone, 7-hydroxycoumarin, ketoconazole, 4-methylpyrazole, omeprazole, paroxetine, phenacetin, quinidine, sibutramine, sulfaphenazole, testosterone, tetraethylthiuram disulfide, ticlopidine, tienilic acid, tolbutamide, tranylcypromine, verapamil, and reduced form of NADPH (as a tetrasodium salt) were purchased from Sigma-Aldrich (St. Louis, MO, USA). Dextrorphan and 4-hydroxytolbutamide were purchased from Toronto Research Chemicals Inc. (North York, ON, Canada). Bupropion, carbamazepine (internal standard, IS), dextromethorphan, fluvoxamine, fluoxetine, and 8-methoxypsoralen were purchased Wako Co. (Tokyo, Japan). All other chemicals and reagents used were of analytical grade.

### 4.2. RI of Sauchinone on CYPs Activities in HLMs

Sauchinone was dissolved in *N*,*N*-dimethyl formamide and serially diluted with acetonitrile. All CYP selective substrates were dissolved and serially diluted with methanol to give a final organic solvent concentration of 1.0% (set A) and 0.5% (set B) in the incubation mixture. Concentrations of CYP selective substrates were used close to their reported Michaelis constant (*K*_m_, substrate concentration at 1/2 of maximum velocity) values [33] and our unpublished data as shown below: 50 μM phenacetin, 5 μM coumarin, 50 μM bupropion, 100 μM tolbutamide, 100 μM *S*-mephenytoin, 5 μM dextromethorphan, 50 μM chlorzoxazone, and 50 μM testosterone (substrates of CYP1A2, 2A6, 2B6, 2C9, 2C19, 2D6, 2E1, and 3A4, respectively). Well-known direct inhibitors on the activities of CYP1A2, 2A6, 2B6, 2C9, 2C19, 2D6, 2E1, and 3A4 were fluvoxamine, tranylcypromine, paroxetine, sulfaphenazole, omeprazole, quinidine, 4-methylpyrazole, and ketoconazole, respectively [49,50]. Then, 100 μL of mixture containing 5 μL of HLM (1 mg protein), 0.1 M sodium phosphate buffer (pH 7.4), 1 or 0.5 μL of substrate cocktail set (set A or B, respectively), and 1 μL of inhibitor (sauchinone or a well-known inhibitor for each CYP isoform at 0–300 μM) was incubated at 37 °C for 5 min in a thermomixer at 500 rpm. The incubation reaction was initiated by adding 2 μL of 50 mM NADPH. It was then incubated at 37 °C for 30 min. The reaction was stopped by adding 50 μL of incubation mixture to 100 μL of ice-cold acetonitrile containing 20 ng/mL IS. The samples were then centrifuged at 4 °C for 10 min at 12,000 rpm. Metabolites of CYP isoforms in supernatants were analyzed by high-performance liquid chromatography-tandem mass spectrometry (HPLC-MS/MS). Half maximal inhibitory concentration (IC_50_) values of sauchinone were estimated for inhibition of each CYP isoform.

### 4.3. K_i_ Values of Sauchinone for CYP2B6, 2C19, 2E1, and 3A4 in HLMs

Based on IC_50_ values, the *K_i_*s of sauchinone for CYP2B6, 2C19, 2E1, and 3A4 were determined. Different concentrations (20, 50, and 200 μM) of sauchinone were used. Other procedures were similar to those mentioned above regarding the reversible inhibitory effects of sauchinone. The inhibitory characteristics of sauchinone were initially estimated by nonlinear least squares regression analysis. Its *K_i_* values were determined by Dixon plots [51].

### 4.4. TDI of Sauchinone on CYP Activities in HLMs

The IC_50_ shift assay is the most recommended method to evaluate TDI of sauchinone efficiently and conveniently. Enzymatic activity changes have been usually detected by comparing the presence or absence of a pre-incubation period with an inhibitor during a defined period. Usually, well-known time-dependent inhibitors on CYPs have showed IC_50_ shifts of ≥1.5-fold with a 30-min pre-incubation (versus 0-min pre-incubation; [29]). This cut-off value of IC_50_ shift was adjusted to identify the TDI of sauchinone in this study. The well-known time-dependent inhibitors of activities of CYP1A2, 2A6, 2B6, 2C9, 2C19, 2D6, 2E1, and 3A4 were furafylline, 8-methoxypsoralen, ticlopidine, tienilic acid, fluoxetine, paroxetine, disulfiram, and verapamil, respectively.

After 100 μL of mixture containing HLM (1 mg protein), 0.1 M sodium phosphate buffer (pH 7.4), 1 or 0.5 μL of substrate cocktail set (set A or set B, respectively), and 1 μL of inhibitor (sauchinone or a well-known inhibitor at 10-fold higher concentration than the final incubation concentration at 0–300 μM) was incubated at 37 °C for 5 min in a thermomixer at 500 rpm, the incubation reaction was initiated by adding 2 μL of 50 mM NADPH. Incubation time was then divided into 0 and 30 min. After inactivation incubation for 0 min or 30 min, 100 μL of the mixture containing a 10 μL aliquot from each inactivation incubation tube, 0.1 M sodium phosphate buffer, 1 or 0.5 μL of substrate cocktail set (set A or B), and 2 μL of 50 mM NADPH was incubated for 10 min. These reactions were stopped by adding 50 μL of mixture to 100 μL of ice-cold acetonitrile containing 20 ng/mL IS. The sample was centrifuged at 4 °C for 10 min at 12,000 rpm. Metabolites of CYP isoforms in supernatants were analyzed by HPLC-MS/MS.

### 4.5. HPLC-MS/MS Analysis of Metabolites from CYP Substrates, Sibutramine, Clopidogrel, and Chlorzoxazone

Metabolites from CYP substrates were analyzed using an API4000 triple quadrupole mass spectrometer HPLC-MS/MS system (Applied Biosystems Sciex, Foster City, CA, USA) in multiple reaction monitoring mode with an electrospray ionization interface for positive ions ([M + H]^+^) and negative ions ([M − H]^−^). Separation was performed on a reversed-phase C_18_ column (Atlantis d-C_18_, 2.1 mm × 150 mm i.d., 3 μm particle size; Waters, Dublin, Ireland) maintained at 30°C. The mobile phase consisted of water containing 0.1% formic acid (A) and acetonitrile (B) at a flow rate of 0.5 mL/min. The following gradient elution program used: (1) mobile phase A was set to 100% at 0 min until 0.1 min; (2) a linear gradient was run to 50% in 4.0 min. It was held at 50% until 6.5 min; (3) a linear gradient was run to 0% in 9.5 min until 14.0 min; (4) a linear gradient was run to 100% in 14.3 min and re-equilibrated for 5.7 min. The total run time was 20 min. Negative-ion mode was used for the analysis of 4-hydroxytolbutamide and 6-hydroxychlorozoxazone.

Turbo ion-spray interface was operated in positive ion mode at an ion source voltage of 5500 V (negative ion mode −4500 V) and a temperature of 500 °C. Operating conditions optimized by flow injection of a mixture of all analytes were: nebulizing gas flow, 50 L/min; turbo ion-spray gas flow, 50 L/min; curtain gas flow, 20 L/min; collision gas (nitrogen) pressure, 5 Torr; declustering potential, 71.0 eV; and entrance potential, 10 eV. The *m*/*z* values (voltage of CE) for acetaminophen, 7-hydroxycoumarin, hydroxybupropion, 4-hydroxytolbutamide, 4-hydroxymephenytoin, dextrorphan, 6-hydroxychlorozoxazone, 6α-hydroxytestosterone, and IS were *m*/*z* 152.087 → 110.100 (CE 21 V), 163.072 → 107.000 (CE 31 V), 257.257 → 239.100 (CE 17 V), 285.016 → 185.800 (CE −26 V), 235.249 → 150.000 (CE 27 V), 258.178 → 157.100 (CE 49 V), 184.868 → 120.800 (CE −40 V), 305.229 → 269.100 (CE 23 V), and 237.193 → 194.100 (CE 25 V), respectively.

For analysis of sibutramine, a mobile phase was made, consisting of 10 mM ammonium acetate adjusted to pH 4 with acetic acid: acetonitrile (94:6, *v*/*v*) and was eluted at 0.2 mL/min. The *m*/*z* values (voltage of CE) for sibutramine and domperidone (IS) were 280.1 → 125.3 (CE 18 V) and 427.3 → 175.3 (CE 19 V), respectively, in positive mode. For analysis of clopidogrel, a mobile phase was consisted of methanol:acetonitrile:distilled water with 0.1% formic acid (75:13:17, *v*/*v*/*v*) and eluted at 0.35 mL/min. The *m*/*z* for clopidogrel and ticlopidine (IS) were 322.0 → 212.2 (CE 18 V) and 264.3 → 154.1 (CE 17 V), respectively, in positive mode. Chlorzoxazone was analyzed in negative mode with the same mobile phase used for CYP specific metabolites. The *m*/*z* for chlorzoxazone and carbamazepine (IS) were 167.5 → 131.6 (CE −26 V) and 237.1 → 194.1 (CE −29 V), respectively. Other parameters of mass spectrometers were the same as those used for analysis of metabolites of CYP substrates.

### 4.6. Data Analysis for IC_50_ Values

For RI and TDI screening of sauchinone, CYP-mediated metabolic activities in the presence of sauchinone or well-known inhibitors were expressed as percentages of the corresponding control values. From percentages of control activity versus inhibitor concentrations, a sigmoid shaped curve was fitted to the data and IC_50_, as an enzyme inhibition parameter, was calculated by fitting the Hill equation to the data using GraphPad Prism 5 (GraphPad Software Inc., San Diego, CA, USA).

### 4.7. Molecular Docking Study

Based on enzyme inhibition results, interactions of sauchinone with human CYP3A4, CYP2B6, 2C19, and CYP2E1 were analyzed by molecular docking studies. X-ray crystal structures of human CYP3A4 (PDB code: 3UA1; [36]), CYP2B6 (PDB code: 3IBD; [9]), CYP2C19 (PDB code: 4GQS; [42]), and CYP2E1 (PDB code: 3GPH; [45]) were retrieved from Research Collaboratory for Structural Bioinformatics (RCSB) Protein Databank (https://www.rcsb.org/). Discovery Studio 2017 R2 (Dassault Systèmes BIOVIA, San Diego, CA, USA) was employed to create the sauchinone structure. It was also used for energy minimization. AutoDock 4.2.6 software (Molecular Graphics Laboratory, La Jolla, CA, USA) was used to perform molecular docking [52]). Both protein and ligand structures were processed prior to molecular docking studies using AutoDock Tools 1.5.6 [53]. For each CYP, the AutoGrid program was used to create a grid box around co-crystallized ligand to cover active site of the protein. Grid of size 40 × 40 × 40 Å^3^ with 0.375 Å spacing was centered on co-crystallized ligand of each CYP. Docking simulations were performed using the Lamarckian genetic algorithm (LGA) [54]. A total of 100 runs along with 25 × 10^5^ energy evaluations and 27,000 iterations were carried out. Default values were used for other parameters. Docked poses were chosen based on scoring functions and protein–ligand interactions. Discovery Studio 2017 R2 was used to visualize binding interactions.

### 4.8. Animals

Protocols for animal studies were approved by the Institutional Animal Care and Use Committee of Dongguk University-Seoul, Seoul, South Korea (2014-DGU-04). Male ICR mice (5 weeks old, weight of 20–25 g) were purchased from the Charles River Company Korea (Orient, Seoul, Korea). All mice were maintained under controlled room temperature, humidity, and light (12/12 h light/dark cycle). They were provided free access to food and water. These mice were allowed to acclimate to these conditions for three days prior to inclusion in the experiments.

### 4.9. Drug Interaction of Sibutramine, Clopidogrel, or Chlorzoxazone with Sauchinone in Mice

To investigate the inhibitory effect of sauchinone on CYP2B6, 2C19, 2E1, or 3A4-mediated metabolism, pharmacokinetic interactions of drugs (sibutramine, clopidogrel, or chlorzoxazone) with sauchinone were examined in mice. Sibutramine, clopidogrel, and chlorzoxazone were chosen as representative drugs mainly metabolized by CYP2B6, 2C19/3A4, and 2E1, respectively [24,25,26,47].

Surgical procedures for mice were conducted under tiletamine HCl and zolazepam HCl anesthesia by intramuscular injection. The carotid artery (for blood sampling) cannulation was carried out using catheters (BASi, West Lafayette, IN, USA). Drug administration and blood sampling were performed at 5 h after surgery [55]. Thirty minutes after oral administration of 100 mg/kg sauchinone, 1 mg/kg sibutramine was administered intraperitoneally to mice. Blood samples were collected via the carotid artery at 0, 5, 15, 30, 60, 90, 120, 240, 360, and 480 min after administration of sibutramine. Ten μL of blood was collected into a micro-vial containing 50 μL of 12.5 units/mL heparinized saline using micro-sampling system. After centrifugation of each micro-vial, 50 μL of plasma with heparinized-saline was collected from the supernatant and analyzed by HPLC-MS/MS. Instead of sibutramine, 10 mg/kg clopidogrel or 10 mg/kg chlorzoxazone was administered thirty minutes after oral administration of 100 mg/kg sauchinone or vehicle. Other procedures were the same as those used for the sibutramine pharmacokinetic study.

After analyzing sibutramine, clopidogrel, or chlorzoxazone concentrations in plasma by HPLC-MS/MS, pharmacokinetic parameters were calculated as follows. AUC was calculated using the trapezoidal rule method. Standard methods [56] were used to calculate pharmacokinetic parameters using non-compartmental analysis (WinNonlin 2.1; Pharmasight Corp., Mountain View, CA, USA). *C*_max_ and *T*_max_ were read reversibly from extrapolated data.

## 5. Conclusions

Our results demonstrated for the first time that sauchinone has potential to inhibit CYP2B6, 2C19, 2E1, and/or 3A4-mediated metabolism noncompetitively. This could cause pharmacokinetic interactions between drugs and sauchinone. The inhibitory effects of sauchinone on CYP2B6, 2E1, and 3A4 were time-dependent. Molecular docking studies identified interacting residues of CYP2B6, 2C19, 2E1, and 3A4 with sauchinone, supporting the inhibitory potential of sauchinone on these CYP isoforms. At preclinical levels, sauchinone inhibited the metabolic clearance of drugs such as sibutramine, clopidogrel, and chlorzoxazone. Sauchinone and drug interactions might cause unwanted effects. Therefore, more attention is needed when using herb–drug combination therapies.

## Figures and Tables

**Table 1:** IC_50_ (μM) values of sauchinone and each well-known reversible inhibitor on respective CYP activity in HLMs.

CYPs	Sauchinone (μM)	Well-Known Inhibitor ^a^ (μM)
CYP1A2	>300	0.0340
CYP2A6	>300	1.99
CYP2B6	0.458 ± 0.0932	0.154
CYP2C9	>300	0.340
CYP2C19	3.60 ± 2.54	6.59
CYP2D6	>300	0.0221
CYP2E1	35.8 ± 50.1	0.519
CYP3A4	0.207 ± 0.193	0.0845

^a^ Fluvoxamine, tranylcypromine, paroxetine, sulfaphenazole, omeprazole, quinidine, 4-methylpyrazole, and ketoconazole are well-known inhibitors of CYP1A2, 2A6, 2B6, 2C9, 2C19, 2D6, 2E1, and 3A4 in RI mode, respectively.

**Table 2:** IC_50_ (μM) values of sauchinone and each well-known reversible inhibitor on the respective CYP activity in HLMs. Also shown are the IC_50_ values and IC_50_ shifts of sauchinone and each well-known time-dependent inhibitor on each CYP’s activity in HLMs.

CYPs	Sauchinone			Well-Known Inhibitor ^a^		
	IC_50_ (μM)		IC_50_ Shift	IC_50_ (μM)		IC_50_ Shift
	0 min	30 min		0 min	30 min	
CYP1A2	>300	>300	-	0.533	0.00424	126
CYP2A6	>300	>300	-	5.07	0.996	5.09
CYP2B6	36.4 ± 27.0	3.93 ± 5.72	9.28	0.529	0.0745	7.10
CYP2C9	>300	>300	-	50.7	0.157	323
CYP2C19	28.5 ± 0.267	19.4 ± 2.92	1.47	0.0312	0.00297	10.5
CYP2D6	>300	>300	-	2.59	0.288	8.99
CYP2E1	105 ± 57.0	5.04 ± 1.91	20.9	6.70	2.16	3.10
CYP3A4	24.5 ± 51.3	1.14 ± 2.29	21.4	20.3	1.61	8.41

^a^ Furafylline, 8-methoxypsoralen, ticlopidine, tienilic acid, fluoxetine, paroxetine, disulfiram, and verapamil are well-known inhibitors of CYP1A2, 2A6, 2B6, 2C9, 2C19, 2D6, 2E1, and 3A4 in TDI mode, respectively.

**Table 3:** Pharmacokinetic parameters of sibutramine, clopidogrel, or chlorzoxazone after its oral administration with or without sauchinone.

Parameters	Sibutramine	Clopidogrel	Chlorzoxazone
Without sauchinone	(*n* = 5)	(*n* = 5)	(*n* = 5)
AUC (μg min/mL)	3980 ± 308	2680 ± 372	2390 ± 446
*C*_max_ (μg/mL)	28.2 ± 2.86	35.8 ± 5.21	28.8 ± 4.76
*T*_max_ (min)	15.0 (15.0−15.0)	15.0 (15.0−15.0)	15.0 (5.00−15.0)
t_1/2_ (min)	197 ± 35.9	131 ± 6.70	73.7 ± 6.34
CL/*F* (mL/min/kg)	0.253 ± 0.0208	3.81 ± 0.514	4.37 ± 0.972
With sauchinone	(*n* = 5)	(*n* = 5)	(*n* = 5)
AUC (μg min/mL)	4920 ± 415 ^a^	3510 ± 288 ^a^	3850 ± 880 ^a^
*C*_max_ (μg/mL)	39.9 ± 4.97 ^a^	42.0 ± 2.33 ^a^	40.5 ± 6.55 ^a^
*T*_max_ (min)	15.0 (15.0−15.0)	15.0 (15.0−15.0)	30.0 (15.0−30.0) ^a^
t_1/2_ (min)	165 ± 31.8	106 ± 25.6	66.8 ± 7.99
CL/*F* (mL/min/kg)	0.205 ± 0.0186 ^a^	2.87 ± 0.225 ^a^	2.71 ± 0.519 ^a^

AUC, area under the plasma concentration–time curve from time zero to the last measured time to infinity; *C*_max_, peak plasma concentration; *T*_max_, time to reach *C*_max_; t_1/2_, terminal half-life; CL/*F*, clearance/absolute bioavailability.; ^a^ Significantly different (*p* < 0.05) compared with that without sauchinone.
